# Maraviroc intensification for HIV-1-positive immunological non-responders (INRs) despite virological suppression during HAART

**DOI:** 10.1186/1758-2652-13-S4-O44

**Published:** 2010-11-08

**Authors:** S Rusconi, P Vitiello, F Adorni, E Colella, E Focà, AF Capetti, P Meraviglia, C Abeli, S Bonora, M D'Annunzio, A Di Biagio, A Di Pietro, L Butini, G Orofino, S Farina, G d'Ettorre, D Francisci, A Soria, AR Buonomini, C Tommasi, MP Trotta, M Capasso, E Merlini, GC Marchetti

**Affiliations:** 1 Dipartimento di Scienze Cliniche “Luigi Sacco”, Sezione di Malattie Infettive-Immunopatologia, Università degli Studi di Milano, via GB Grassi, 74, Milano, Italy; 2 ITB-CNR, Segrate (MI), Italy; 3 Clinica Malattie Infettive, Università degli Studi di Brescia, Spedali Civili, Brescia, Italy; 4 1a Div. Malattie Infettive, Ospedale Luigi Sacco, Milano, Italy; 5 2a Div. Malattie Infettive, Ospedale Luigi Sacco, Milano, Italy; 6 Div. Malattie Infettive, Ospedale di Circolo, Busto Arsizio (VA), Italy; 7 Clinica Malattie Infettive, Università degli Studi di Torino, Torino, Italy; 8 Clinica di Malattie Infettive, Ospedale Policlinico di Bari, Bari, Italy; 9 Clinica Malattie Infettive, Università degli Studi di Genova, Ospedale San Martino, Genova, Italy; 10 Div. Malattie Infettive, Ospedale S. Maria Annunziata, Antella (FI), Italy; 11 Servizio Regionale di Immunologia Clinica e Tipizzazione Tessutale, Università Politecnica delle Marche, Torrette di Ancona (AN), Italy; 12 Div. A Malattie Infettive, Ospedale Amedeo di Savoia, Torino, Italy; 13 Istituto di Clinica Malattie Infettive, Università Cattolica del Sacro Cuore, Roma, Italy; 14 Div. Malattie Infettive, Ospedale Policlinico Umberto I, Roma, Italy; 15 Clinica di Malattie Infettive, Ospedale S. Maria della Misericordia, Perugia, Italy; 16 Div. Malattie Infettive, Ospedale San Gerardo, Monza, Italy; 17 Clinica Malattie Infettive, Università Tor Vergata, Roma, Italy; 18 INMI “Lazzaro Spallanzani”, IV Div. Malattie Infettive, Roma, Italy; 19 INMI “Lazzaro Spallanzani”, III Div. Malattie Infettive, Roma, Italy; 20 Clinica Malattie Infettive e Tropicali, Università degli Studi di Milano, Ospedale San Paolo, Milano, Italy

## Purpose

15-30% of HAART-treated HIV-1-positive patients (pts) lack CD4+ increase despite full HIV viremia suppression. The increased risk for INR to progress till AIDS led us to investigate maraviroc (MVC) as a tool to intensify HAART in terms of immunological recovery.

## Methods

Randomised, multicentre, proof-of-concept study enrolling 100 pts divided into 2 arms (1:1), A:HAART+MVC, B:HAART. Inclusion criteria were: CD4 count ≥200 cells/µL and/or a recovery of CD4 cells <25% compared to the HAART initiation and with a stable virologic suppression after 1 year of HAART. Ultrasensitive HIV-RNA was quantified via Amplicor HIV-1 Monitor Kit v1.5. Naive CD45RA+, memory CD45RA-, activated HLA-DR+CD38+, proliferating Ki67+, CD4+, CD8+ T-cells were measured by flow cytometry. T-test was used for intra and inter-group comparisons.

## Results

100 pts have been randomized 64 pts reached week(w) 12: 37 in A and 27 in B arms. At baseline (BL), CD4/CD8 and immune-phenotype were comparable in arm A and B. At w12 no significant changes in mean CD4 recovery (+41.9 vs +24.5/µL; p=.241) and a statistically significant change in mean CD8+ count (+164.2 vs -27.3/µL; p=.004) were observed between pts in arm A and B.

At BL and w12 an immunological study was carried out in 24 pts (13:arm A, 11:arm B): at w12, while B pts experienced a contraction of naïve CD4 (81 to 67%; p=.02) and CD8 (81 to 77%; p=.04) with a parallel rise in memory CD4 (16 to 30%; p=.02) and CD8 (13 to 17%; p=.06), no significant loss of naive CD4 (70 to 57%; p=.18) and CD8 (69 to 66%; p=.42) was displayed by A pts with a tendency to higher gain in memory CD4 (24 to 40%; p=.06) and CD8 (11 to 25%; p=.008). By w12, a similar reduction in activated HLA-DR+CD38+ CD8 and CD4 was shown in B (p=.05) and A pts (p=.03 and p=.02 for CD8 and CD4). A trend to Ki67+CD8 reduction was shown in A (p=.06) and not in B pts (p=.45). HIV-RNA quantification evidenced a trend to higher median values (BL vs w12) in B pts: 2 vs 5 cp/mL (p=.37).

## Conclusions

MVC does not seem to increase CD4 amount at significant level compared to arm B. Treatment with MVC is associated with a significant CD8+ gain, a preservation of phenotypically naïve CD4+ and parallel rise of memory pool, suggesting a role of MVC in reducing peripheral antigen-driven T-cell death, possibly preserving new T-cell production. MVC is able to further reduce T-cell activation and proliferation, suggesting a possible influence in better controlling the pro-inflammatory status.

We acknowledge the participation of all investigators of the HSL/MVC01/2008 Study Group (NCT00884858).

